# Shift Work, Psychological Health Disorders, and Job Security Among Nurses: A Cross-Sectional Study

**DOI:** 10.3390/healthcare13030221

**Published:** 2025-01-22

**Authors:** Rokaya Alghamdi, Ghareeb Bahari

**Affiliations:** 1 Medical Surgical Department, College of Nursing, King Saud University, Riyadh 11421, Saudi Arabia; rokayaalghamdi@gmail.com; 2 Nursing Administration and Education Department, College of Nursing, King Saud University, Riyadh 11421, Saudi Arabia

**Keywords:** nursing, shift work, mental health, job security

## Abstract

Background/Objectives: Shift work is prevalent among nurses, often leading to adverse psychological effects, such as fatigue, depression, anxiety, and stress. Understanding how shift work contributes to psychological health disorders can help healthcare organizations identify critical areas where support should be offered. This study aimed to determine the relationships between shift work, psychological health disorders, and job security among nurses in Saudi Arabia. Methods: This cross-sectional study included 163 nurses, recruited via convenience sampling. The participants completed an online questionnaire that assessed demographic variables, psychological disorders, fatigue, and job security. The data were analyzed using descriptive statistics as well as bivariate analyses to explore relationships between variables. Results: Most participants (73%) worked 12 h shifts, and 67.5% reported fair sleep quality. We found significant correlations among shift work, fatigue, and common psychological disorders. Significant differences were also observed for fatigue (*p* = 0.007) and depression (*p* = 0.008). Both nationality (*p* < 0.001) and shift work (*p* = 0.015) were correlated with anxiety. Similarly, significant differences were found for nationality (*p* = 0.001) and shift work (*p* = 0.002) regarding stress. Conclusions: These findings underscore the psychological challenges faced by nurses related to shift work, emphasizing the importance of addressing fatigue and mental health. Healthcare organizations should implement strategies to enhance job security and support nurses’ well-being to ultimately improve both nurse satisfaction and patient care outcomes. Further research is warranted to explore effective interventions and the long-term effects of shiftwork on nursing professionals.

## 1. Introduction

Shift work is a common practice in the healthcare sector—particularly among nurses who often face rotating schedules that include long day and night shifts [1]. This work structure is essential for providing continuous patient care; however, it can lead to significant challenges for nurses. Shift types typically include fixed, rotating, and split—all of which must be scheduled fairly to reduce burnout [2]. The length of shifts (e.g., 8, 10, or 12 h) should facilitate adequate handover and ensure timely patient care. Prioritizing staff well-being is essential, along with strategies for fatigue management, mental health support, and flexible scheduling [3]. Training programs can help staff adapt to shift work, while technologies, such as scheduling software and telehealth services, can improve efficiency. Finally, compliance with labor laws and union agreements is crucial for creating a supportive work environment [4].

The influence of irregular shift work hours on nurses’ psychological health is critical for healthcare systems. Research demonstrated that fatigue, anxiety, depression, stress, and sleep disturbances are prevalent among nurses working non-traditional hours [5]. Such psychological disorders not only threaten individual well-being but also compromise patients’ quality of care, as stressed and fatigued nurses may experience diminished cognitive function, leading to impaired decision-making and focus [6]. This highlights the need for comprehensive support systems, such as mental health resources and flexible scheduling, to mitigate these challenges. Moreover, providing access to such resources could empower nurses to prioritize their mental health, ultimately benefiting both their own lives and patient care.

Moreover, job security has been linked to nurses’ mental health status. Evidence shows that when nurses feel secure in their positions, they report significantly lower levels of stress and anxiety [7]. Job security would also allow nurses to concentrate more effectively on their duties, leading to improved performance and a stable work environment. Conversely, job insecurity exacerbates mental health issues, as fears about employment stability can lead to chronic stress [8]. This relationship highlights the importance of fostering a stable work environment where nurses can concentrate on their clinical responsibilities rather than worry about potential layoffs or departmental changes [9]. Enhancing job security not only supports nurses’ mental health but also translates into improved patient care, as secure and engaged employees are more likely to deliver high-quality services [6].

In some countries, such as Saudi Arabia, the healthcare system includes public and private healthcare settings, providing different options for citizens and residents. The public sector is government-funded and widely accessible, while private hospitals provide shorter wait times, thereby enhancing overall healthcare delivery. The local nursing shift system in healthcare facilities is characterized by a rotating schedule that typically includes both day and night shifts, often structured in 12 h cycles to ensure continuous patient care. This system is essential for meeting the demands of a 24/7 healthcare environment, particularly in hospitals where the patient needs are constant and diverse [10]. While the rotating shift system helps maintain adequate staffing levels, it can also pose multiple physical and psychological challenges [11]. To address these issues, several healthcare organizations in Saudi Arabia are implementing strategies aimed at supporting healthcare professionals’ mental health and well-being—including flexible scheduling options, wellness programs, and access to mental health resources.

The relationships between shift work, psychological health disorders, and job security are complex. Different shift schedules can impact employees, particularly in sectors like healthcare, as they may lead to sleep disturbances and circadian rhythm issues, contributing to psychological problems and ultimately resulting in feelings of insecurity or dissatisfaction. These varying shifts can also cause employees to experience social isolation due to missed social interactions, which can further affect their mental health. Additionally, job insecurity or dissatisfaction can lead to chronic psychological strain that significantly impacts patient care and safety [12]. Understanding how shift work contributes to psychological health disorders can help healthcare organizations identify critical areas for interventions and support [13]. In addition, exploring the role of job security in this context can provide insights into the creation of a more stable and supportive work environment for nurses [14]. Therefore, this study aimed to determine the relationships between shift work, psychological health disorders, and job security among nurses in Saudi Arabia. The following hypotheses were formed:

**Hypothesis** **1:** *Common psychological disorders such as depression, anxiety, and stress are likely to be high among nurses due to long-shift systems*.

**Hypothesis** **2:** *There may be significant associations between common psychological health disorders*.

**Hypothesis** **3:** *Specific demographic variables may have significant differences in the means of some psychological health disorders*.

## 2. Materials and Methods

### 2.1. Study Design and Setting

This was a cross-sectional, descriptive-correlational study conducted on nurses working in the Riyadh region of Saudi Arabia. Riyadh is the capital of the country and is known for its exceptional medical services and advanced facilities that provide high-quality patient care. Approximately 8 million people reside in Riyadh, thus necessitating the existence of multiple healthcare facilities capable of addressing diverse medical requirements. The STROBE checklist was used to ensure that this study adhered to best practices in research [15] (see Supplementary Materials).

### 2.2. Sampling Process

A convenience sampling approach was selected because of its ease of access and availability [16]. Nurses who had been working in the Riyadh region for at least one year were included. Those with limited levels of English proficiency were excluded to reduce potential misunderstandings of the questionnaire. Nurses who chose not to participate, as well as pregnant nurses, were also excluded because they were unlikely to be working long shifts during their pregnancies. G*Power V.3.1 (Heinrich-Heine-Universität, Düsseldorf, Germany) was used to estimate that the sample size needed for this study was 143 participants. An additional buffer of ~10% was recruited to compensate for any missing data, resulting in a minimum sample of 157 subjects.

### 2.3. Instrumentation

This study used three instruments: demographic variables, common psychological disorders, and job security.

### 2.4. Demographic Variables

The demographic data included age, gender, marital status, nationality, educational level, income level, shift work, department, distance to the hospital, employment contract, and years of experience.

### 2.5. Common Psychological Disorders

The second part of the questionnaire included three sections regarding the psychological impact of shift work. Section one involved the Depression Anxiety Stress Scale (DASS), version 21 (DASS-21), a self-reported questionnaire consisting of three subscales, each with 7 items [17]. The participants rated their symptoms on a 4-point Likert scale ranging between 0 (does not apply at all) and 3 (applies to a great extent). The scores were categorized as normal, mild, moderate, severe, or extremely severe [17]. Cronbach’s alpha for the DASS-21 was reported at an excellent level [18]. The second section included the Fatigue Scale, which assessed fatigue through a self-reported questionnaire containing 10 statements [19]. Participants chose one of the five categories (1: never; 2: sometimes; 3: regularly; 4: often; 5: always) for each statement. The total score for fatigue ranged between 10 and 50, with total scores of >22 indicating fatigue. The fatigue-related subscales demonstrated a strong psychometric value of 0.90. Four of the items exhibited gender-based biases, with women scoring higher than men; however, the adjusted scores indicated a negligible effect on the individual scores [19]. The third section involved a single item evaluating nurses’ quality of sleep over the preceding 7 days and how it influences their ability to perform well at work [20].

### 2.6. Job Security

Due to a lack of access to existing instruments, a closed-ended question was posed to the nurses about the level of security they felt at work. This item is a categorical measure that includes the following responses: (1) I feel insecure at work; (2) I rarely feel secure at work; (3) I somewhat feel secure at work; and (4) I feel completely secure at work. A score of 4 indicates a complete feeling of security at work.

### 2.7. Data Collection Procedures

An online questionnaire, available in English, was created using Google Forms and shared with this study sample by their managers. A QR code was also provided to facilitate access to this study questionnaire. Personal references further helped in accessing some participants. These combined efforts maximized participation and enhanced the quality of the data collected. The estimated time to complete the questionnaire was ~20 min.

### 2.8. Data Analysis

IBM SPSS Statistics for Windows, version 28 (IBM Corp., Armonk, NY, USA), was used to analyze the collected data. Missing data were handled using mean imputation for continuous variables or mode imputation for categorical variables [21]. Descriptive statistics were run to assess both continuous and categorical variables. Bivariate analyses, including independent-sample *t*-tests and one-way analysis of variance (ANOVA), were conducted to determine significant differences among groups after verifying their assumptions. Pearson’s correlation coefficient was also used to examine the associations between continuous variables. The significance level was set at 95%, and Cronbach’s alpha was used to assess the reliability of the structured questionnaire.

### 2.9. Ethical Approval

Institutional review board approval was obtained prior to data collection. In the initial section of the questionnaire, a clear note detailing the purpose and content of this study was provided, along with an option for the participants to indicate voluntary participation. The participants’ responses were treated confidentially, and the electronic questionnaire was securely distributed through authorized channels, such as the hospital’s research center, nursing managers, and personal references.

## 3. Results

### 3.1. Descriptive Findings

This study ultimately included 163 participants (Table 1). Most of the included nurses were female (84.7%), with a significant percentage being non-Saudi nationals (63.2%). The average participant age was 34.81 years (standard deviation = 6.25; range = 23–60 years). Approximately half were unmarried (50.9%), and the vast majority held a bachelor’s degree (80.4%). A high proportion reported an income level of ≥SR 7000 (77.9%), and most were employed on a contractual basis (73.6%). More than half of the nurses commuted for 30 min to 1 h daily to reach the hospital (53.4%). Nearly all had >2 years of experience in nursing (93.3%), and the majority (73%) worked under a 12 h shift system across various departments.

Most of the participants (67.5%) reported having fair sleep quality, while 20.3% indicated poor to terrible sleep quality. In terms of feeling secure at work, most of the participants (50.9%) reported feeling somewhat secure in their workplace. The participants exhibited a mean fatigue level of 21.06 (standard deviation, 6.11; range, 10–43). Regarding depression, the average level was 10.87 (standard deviation, 3.68; range, 7–23). The average anxiety level among the participants was 10.61 (standard deviation, 3.68; range, 7–22). The average stress level was 10.29 (standard deviation, 4.02; range, 7–21). Cronbach’s alpha was determined to be 0.94, indicating an excellent level of reliability.

### 3.2. Bivariate Analyses

#### 3.2.1. Pearson’s Correlations

Pearson’s correlation analysis indicated that fatigue was positively associated with depression (r = 0.433, *p* < 0.001), anxiety (r = 0.418, *p* < 0.001), and stress (r = 0.497, *p* < 0.001). Depression was also associated with both anxiety (r = 0.683, *p* < 0.001) and stress (r = 0.692, *p* < 0.001). Anxiety was also determined to be associated with stress (r = 0.828, *p* < 0.001). No significant associations were found between age and these variables (*p* > 0.05) (Table 2).

#### 3.2.2. Independent-Sample *t*-Tests

Our independent-sample *t*-tests revealed significant differences in the means of shift work and fatigue (*p* = 0.007), as well as depression (*p* = 0.008). Notable differences were also observed in the means of both nationality (*p* < 0.001) and shift work (*p* = 0.015) in relation to anxiety. Similarly, significant differences were found in the means of nationality (*p* = 0.001) and shift work (*p* = 0.002) with respect to stress levels. More details regarding the differences in nationalities, work shifts, and main variables are presented in Table 3 and Table 4.

#### 3.2.3. One-Way ANOVA Findings

A statistically significant difference was found between the work department and fatigue (F [4, 162] = 3.169, *p* = 0.015), following one-way ANOVA. A similar process also revealed statistically significant differences for education (F [2, 162] = 4.766, *p* = 0.010), sleep quality (F [4, 162] = 3.483, *p* = 0.009), and job security (F [3, 162] = 3.689, *p* = 0.013) in relation to depression. Moreover, statistically significant differences were reported for education (F [2, 162] = 7.656, *p* < 0.001) and sleep quality (F [4, 162] = 2.462, *p* = 0.047) and their associations with anxiety. Statistically significant differences were also found for education (F [2, 162] = 11.992, *p* < 0.001) and sleep quality (F [4, 162] = 3.440, *p* < 0.010) regarding stress levels. See Table 5 for more details.

## 4. Discussion

This study examined the relationships among shift work, common psychological health disorders, and job security among nurses working in Saudi Arabia. Significant findings were reported that shed light on the main variables of this study.

The findings of our bivariate analyses are consistent with the previous international literature, wherein studies have confirmed positive relationships between fatigue and psychological disorders—including depression, anxiety, and stress. This aligns with the research conducted by Al Maqbali et al., who similarly found significant associations between fatigue and high levels of depression and anxiety [22]. Garbarino and Magnavita highlighted that fatigue is linked to stress, particularly in shift workers who experience increased instances of physical and emotional fatigue caused by unpredictable working hours [23]. Though these findings are expected to be available due to their nature and availability in one questionnaire, they still suggest that fatigue can be considered a significant predisposing factor towards the development and exacerbation of psychological disorders, thereby supporting our understanding of occupational health hazards in the healthcare sector.

However, in this study, the correlation between anxiety and stress was notably stronger than what has been previously reported. Unlike earlier studies that treated these variables as independent but interrelated psychological indicators [24,25], our results revealed a stronger interdependence between anxiety and stress among Saudi nurses, reflecting the unique context of nursing in Saudi Arabia. This heightened correlation may stem from cultural or environmental factors, such as the high levels of work-related stress present in many Saudi healthcare facilities. Furthermore, consistent with previous research [26], this study demonstrated a significant correlation between depressive symptoms, anxiety, and stress. However, the moderate correlation observed may be attributable to the specific job demands placed on Middle Eastern nurses, as well as cultural expectations that may exacerbate psychological distress.

The current study found that nurses who worked 8 h shifts experienced higher levels of fatigue than those who worked 12 h shifts, which conflicts with the findings of other related studies. For example, researchers found that shift duration was inversely correlated with fatigue levels and staff safety in the healthcare sector [27]. Saville et al. confirmed that employees working 12 h shifts exhibited higher levels of burnout and fatigue than those who worked shorter shifts [28]. Alternate shift schedules could also induce fatigue as a result of constant rotations and limited breaks between shifts, potentially explaining the levels of fatigue observed among the nurses in this study who reported working 8 h shifts [29]. Researchers have also highlighted that individual and environmental factors, such as shift intensity and rest breaks, play key roles in terms of mediating the relationship between shift length and fatigue [30]. This indicates that factors beyond simply shift duration may have contributed to the levels of fatigue reported in this study.

The discrepancy observed between shiftwork patterns and depression, where nurses working 8-h shifts exhibited higher levels of depression than those working 12 h shifts, aligns somewhat with certain previous studies, while also presenting certain variations. For example, Al Maqbali et al. highlighted that longer shift hours could negatively affect fatigue and stress, potentially leading to higher rates of depression [22]. Most global studies have examined various shift lengths without specifically comparing 8 h and 12 h shifts. Conversely, the results of this study suggest that shorter shift durations and lighter work weeks, with more frequent shorter shifts, are linked to increased depressive symptoms. This may be because of disruptions to routines and fewer breaks. This contrasts with the notion that longer shifts are inherently more harmful, indicating that the psychological impacts of shift work may be influenced by other variables, such as workload, job demands, and individual coping strategies. These factors, therefore, merit further exploration in diverse healthcare settings.

Saudi nurses have been reported to experience slightly higher stress and anxiety levels than non-Saudi nurses. In this study, those who worked 8 h shifts had also higher stress and anxiety levels than those working 12 h shifts. The 12 h shifts were associated with depressive symptoms, emotional burnout, and exhaustion in healthcare workers, as a result of extended work hours and limited rest breaks [31]. These studies suggest that long working hours contribute to high anxiety and stress, leading to declines in mental health caused by heightened workloads and inadequate rest time. Conversely, frequent shift changes in short shifts may have a more detrimental impact on mental health by disrupting normal circadian rhythms and quality of rest—potentially explaining the higher levels of anxiety and stress among nurses who work this type of schedule. Factors, such as workload, job satisfaction, and individual characteristics, may also interact with shift length to influence stress and anxiety levels [32].

Consistent with other international studies, our one-way ANOVA results regarding the work department indicated a significant relationship with fatigue. Al Maqbali et al. have demonstrated that nurses in demanding and high-stress departments, such as emergency and intensive care units, experience higher levels of fatigue than those in outpatient and administrative departments [22]. This difference in fatigue levels may be attributable to factors, such as physical and emotional stress, decision-making pressures, and the high patient turnover normally prevalent in these departments. Similarly, nurses in general wards and non-specialist departments suffer from severe fatigue caused by chronic staff shortages and demanding shift schedules [33]. Our present results align with this body of research by highlighting distinct variations in fatigue rates across different work departments and suggesting that factors, such as workload, patient acuity, and staffing ratios, may contribute to these differences in fatigue levels. This underscores the context-specific stressors present in different research environments and emphasizes the unique challenges that healthcare professionals face in their work settings.

Our one-way ANOVA results indicated significant differences in educational level, sleep quality, job security, and depression. After reviewing the literature, we discovered that depression tends to decrease with higher educational levels. Studies have shown that individuals with post-secondary education experience better mental health than those without. For example, education-linked employment improves job circumstances, enhances coping resources, and reduces the prevalence of mental health issues [12]. This is often attributed to factors, such as job control, high income, self-scheduling, and self-pacing, which serve as buffers against stress and contribute to improved psychological well-being. A number of studies have supported the association between sleep quality and depression. Poor sleep quality has been reported to correlate significantly with increased levels of depression [34]. Insufficient sleep has been linked to mood disorders and overall mental health outcomes—a correlation that was also evident in our present findings. The impact of job insecurity on depression may vary depending on geographical and cultural factors. Job insecurity represents a significant predictor of depression [35], owing to the stress and mental health implications of potential job loss.

The statistically significant differences found among education, sleep quality, and anxiety aligned with the findings of other related studies. For example, authors found that education is associated with anxiety, with lower educational levels often leading to increased mental health disorders perhaps caused by limited job opportunities and job insecurity [36]. This discrepancy may be attributable to specific contextual factors within the healthcare environment that may have a more pronounced impact on anxiety in the healthcare setting than those in other settings, thereby accounting for the differences we observed. To further explore these findings, future studies might consider delving deeper into these contextual factors to better understand how educational level, job security, and anxiety are interconnected across different work environments. The significant differences observed in education, sleep quality, and stress in this study are consistent with previous research findings. Research has demonstrated that individuals with lower educational levels often experience higher levels of stress [37], primarily as a result of factors, such as job insecurity and limited opportunities for career advancement. Job insecurity has been identified as a significant stressor, with research indicating that individuals who face it tend to experience elevated stress levels [38].

### 4.1. Implications

Our findings encourage nurse leaders to consider offering sufficient incentives to improve nurses’ mental health and job security. To achieve this, we recommend enhancing organizational awareness of the impact of working shifts and the development of psychological disorders in nurses. Creating and implementing institution-wide regulations to reduce and prevent psychological disorders, along with implementing risk management programs at the unit level, are essential for reducing psychological disorders in nurses. Continuing education programs for nurses concerned with coping mechanisms should also be developed, along with teaching preventive measures to define a psychological intervention plan within a mandatory occupational health surveillance program. Finally, we recommend that constructive planning and necessary supportive measures be implemented to protect nurses and minimize their development of psychological disorders to ensure high-quality and safe nursing care.

### 4.2. Limitations

A significant limitation of this study is the inability to generalize the results due to the highly heterogeneous nature of this study population. Further limitations include the cross-sectional design, which may make it difficult to establish causal relationships between variables. Additionally, convenience sampling may result in a sample that is not representative of the entire population. Future research should include a more diverse and representative sample, as well as provide clearer demographic details to enhance the applicability of the findings. It is also suggested to examine different shift types and their interaction with specific demographic variables, and how this interaction may influence individuals’ experiences and outcomes in work settings.

## 5. Conclusions

This study identified significant associations among fatigue, depression, anxiety, and stress. Nurses who worked 8 h shifts reported higher levels of fatigue, anxiety, and depression than those who worked 12 h shifts. These findings underscore the importance of considering shift length in relation to mental health and job-related outcomes in nursing practice. Such insights may also better inform staffing and scheduling decisions to optimize both nurses’ well-being and patient outcomes. Future research should explore additional strategies for managing nurses’ mental health to further improve nursing quality and enhance overall well-being in this demographic.

## Figures and Tables

**Table 1:** Participant characteristics (N = 163).

Characteristics	Frequency	(%)
Gender		
Female	138	(84.7)
Male	25	(15.3)
Nationality		
Non-Saudi	103	(63.2)
Saudi	60	(36.8)
Marital status		
Not married	83	(50.9)
Married	80	(49.1)
Education level		
Diploma	19	(11.7)
Bachelor’s degree	131	(80.4)
Higher education	13	(8.0)
Income		
<Saudi Riyal 7000	36	(22.1)
≥Saudi Riyal 7000	127	(77.9)
Employment contract		
Temporary (on contract)	120	(73.6)
Permanent	43	(26.4)
Distance from hospital		
Few minutes	66	(40.5)
~30–60 min	87	(53.4)
>1 h	10	(6.1)
Years of experience		
<2 years	11	(6.7)
≥2 years	152	(93.3)
Shift type		
8-h shift (Regular)	44	(27.0)
12-h shift (Irregular)	119	(73.0)
Department		
Cardiac Center	15	(9.2)
Oncology center	26	(16.0)
Critical area	36	(22.1)
Inpatient area	36	(22.1)
Other departments	50	(30.7)
Sleep quality		
Terrible	6	(3.7)
Poor	27	(16.6)
Fair	110	(67.5)
Good	19	(11.7)
Excellent	1	(0.6)
Feel at work		
I feel insecure at work	20	(12.3)
I feel rarely secure at work	45	(27.6)
I feel somewhat secure at work	83	(50.9)
I feel completely secure at work	15	(9.2)

**Table 2:** Correlations between continuous variables.

	Age	Fatigue	Depression	Anxiety	Stress
Age	1				
Fatigue	−0.011	1			
Depression	−0.027	0.433 *	1		
Anxiety	−0.028	0.418 *	0.683 *	1	
Stress	−0.052	0.497 *	0.692 *	0.828 *	1

* *p*-value < 0.05.

**Table 3:** *t*-test results by shift type.

	Shift Type	Mean	Std. Deviation	Std. Error Mean	*p*-Value
Fatigue	8-h (Regular)	22.68	7.70313	1.16129	*p* = 0.007 *
	12-h (Irregular)	20.46	5.31992	0.48768	
Depression	8-h (Regular)	12.22	4.45580	0.67174	*p* = 0.008 *
	12-h (Irregular)	10.37	3.23380	0.29644	
Anxiety	8-h (Regular)	12.06	4.17299	0.62910	*p* = 0.015 *
	12-h (Irregular)	10.07	3.35514	0.30756	
Stress	8-h (Regular)	11.81	4.63191	0.69829	*p* = 0.002 *
	12-h (Irregular)	9.73	3.63527	0.33324	

* *p*-value < 0.05.

**Table 4:** *t*-test results by nationality.

	Nationality	Mean	Std. Deviation	Std. Error Mean	*p*-Value
Fatigue	Non-Saudi	20.78	5.67	0.55869	*p* = 0.493
	Saudi	21.53	6.82	0.88121	
Depression	Non-Saudi	10.27	3.34	0.32946	*p* = 0.327
	Saudi	11.91	4.02	0.51928	
Anxiety	Non-Saudi	10.00	3.23127	0.31839	*p* < 0.001 *
	Saudi	11.65	4.19372	0.54141	
Stress	Non-Saudi	9.49	3.52546	0.34737	*p* = 0.001 *
	Saudi	11.66	4.46328	0.57621	

* *p*-value < 0.05.

**Table 5:** Main variables and demographic variables using one-way ANOVA.

	Variable	Categories	Mean	SD	F Value	*p*-Value
Fatigue	Education level	Diploma	24.10	5.05	2.855	0.060
		Bachelor’s degree	20.58	6.30		
		Higher education	21.46	4.15		
	Distance from hospital	Few minutes	21.78	5.82	0.842	0.433
		~30–60 min	20.49	6.43		
		>1 h	21.20	4.91		
	Work department	Cardiac center	19.00	2.29	3.169	0.015 *
		Oncology center	19.30	4.43		
		Critical area	23.13	7.31		
		Inpatient area	22.63	5.91		
		Other departments	19.96	6.28		
	Sleep quality	Terrible	25.83	8.30	1.310	0.268
		Poor	21.70	6.49		
		Fair	20.71	6.08		
		Good	20.36	4.59		
		Excellent	26.00	0.00		
	Job security	I feel insecure at work	21.80	5.36	0.545	0.652
		I feel rarely secure at work	21.48	6.85		
		I feel somewhat secure at work	20.95	6.13		
		I feel completely secure at work	19.40	4.54		
Depression	Education level	Diploma	12.42	4.27	4.766	0.010 *
		Bachelor’s degree	10.45	3.53		
		Higher education	12.92	3.14		
	Distance from hospital	Few minutes	10.60	3.51	2.400	0.094
		~30–60 min	10.80	3.64		
		>1 h	13.30	4.52		
	Work department	Cardiac center	10.53	3.54	0.123	0.974
		Oncology center	10.53	3.12		
		Critical area	11.08	4.21		
		Inpatient area	10.97	3.52		
		Other departments	10.94	3.81		
	Sleep quality	Terrible	15.16	4.87	3.483	0.009 *
		Poor	11.96	3.77		
		Fair	10.45	3.38		
		Good	10.63	3.94		
		Excellent	7.00	0.00		
	Job security	I feel insecure at work	10.55	4.27	3.689	0.013 *
		I feel rarely secure at work	12.11	3.83		
		I feel somewhat secure at work	10.67	3.49		
		I feel completely secure at work	8.73	2.05		
Anxiety	Education level	Diploma	12.78	3.82	7.656	<0.001 *
		Bachelor’s degree	10.07	3.37		
		Higher education	12.84	4.70		
	Distance from hospital	Few minutes	10.84	3.20	1.794	0.170
		~30–60 min	10.22	3.96		
		>1 h	12.40	3.83		
	Work department	Cardiac center	10.40	3.58	0.730	0.573
		Oncology center	9.57	3.21		
		Critical area	10.91	4.22		
		Inpatient area	11.08	4.00		
		Other departments	10.66	3.32		
	Sleep quality	Terrible	13.50	3.27	2.462	0.047 *
		Poor	11.44	4.92		
		Fair	10.10	3.15		
		Good	11.63	4.12		
		Excellent	7.00	0.00		
	Job security	I feel insecure at work	9.40	3.51	1.924	0.128
		I feel rarely secure at work	11.57	4.22		
		I feel somewhat secure at work	10.48	3.55		
		I feel completely secure at work	10.06	2.25		
Stress	Education level	Diploma	13.05	4.91	11.992	<0.001 *
		Bachelor’s degree	9.58	3.59		
		Higher education	13.46	3.73		
	Distance from hospital	Few minutes	10.15	3.51	1.330	0.267
		~30–60 min	10.17	4.27		
		>1 h	12.30	4.80		
	Work department	Cardiac center	10.13	3.52	0.793	0.532
		Oncology center	9.30	3.49		
		Critical area	10.97	4.43		
		Inpatient area	10.72	4.00		
		Other departments	10.06	4.14		
	Sleep quality	Terrible	13.50	5.00	3.440	0.010 *
		Poor	12.03	4.97		
		Fair	9.60	3.49		
		Good	10.94	4.14		
		Excellent	8.00	0.00		
	Job security	I feel insecure at work	9.75	3.94	1.584	0.195
		I feel rarely secure at work	11.15	4.48		
		I feel somewhat secure at work	10.24	3.97		
		I feel completely secure at work	8.73	2.21		

* *p*-value < 0.05; SD: standard deviation.

## Data Availability

The datasets generated and analyzed during the current study are not publicly available due to privacy and ethical restrictions but are available from the corresponding author upon reasonable request.

## Supplementary Materials

The following supporting information can be downloaded at: https://www.mdpi.com/article/10.3390/healthcare13030221/s1.
